# Supplementary material for: Matching Heterogeneous Cohorts by Projected Principal Components Reveals Two Novel Alzheimer’s Disease-Associated Genes in the Hispanic Population
Source: medRxiv. 2025 Jan 19:2025.01.18.25320774. Preprint. [Version 1] doi: 10.1101/2025.01.18.25320774 (PMC11759617; doi:10.1101/2025.01.18.25320774)
Supplement: Supplementary file 1 [file media-1.pdf]

A. NIAGADS HISP

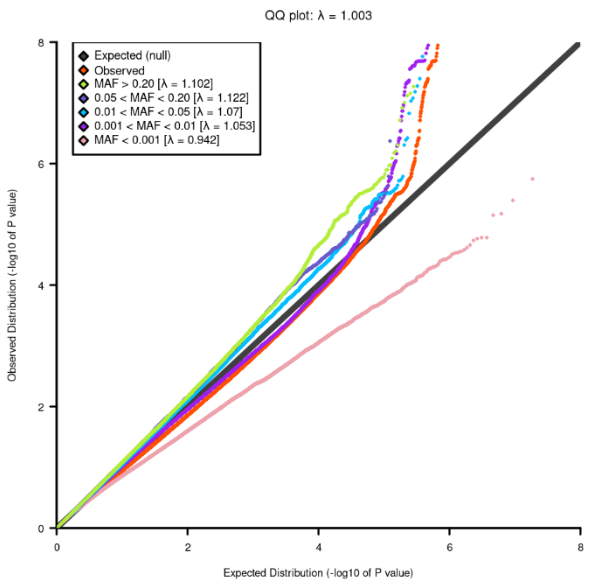

B. AoU Matched using Genetic PCs

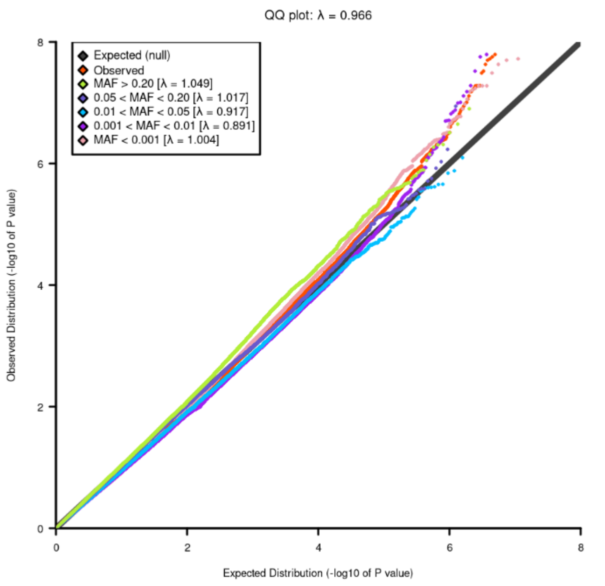

C. AoU Matched using Genetic PCs, Age, and Sex

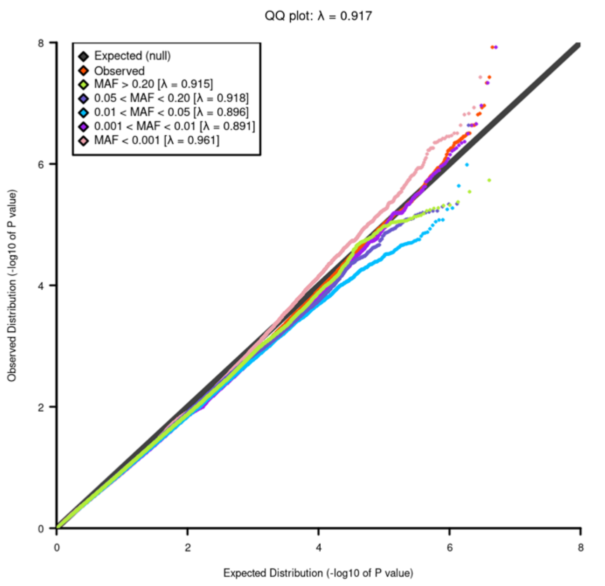

D. Meta-analysis of NIAGADS HISP and AoU Matched using Genetic PCs

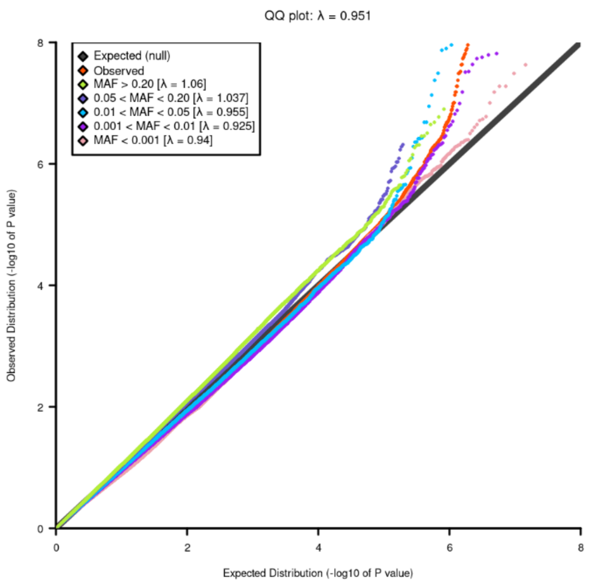

E. Meta-analysis of NIAGADS HISP and AoU Matched using Genetic PCs, Age, and Sex

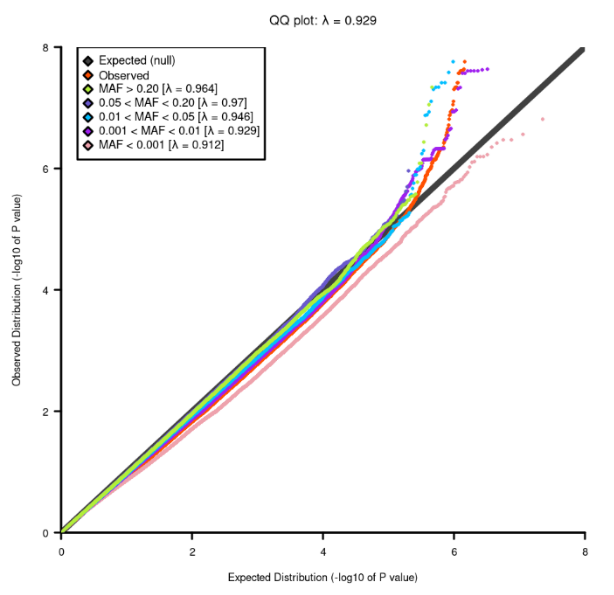

Supplemental Figure 1. QQ plots, with genomic inflation factor, for each dataset.
