# Supplementary material for: Matching Heterogeneous Cohorts by Projected Principal Components Reveals Two Novel Alzheimer’s Disease-Associated Genes in the Hispanic Population
Source: medRxiv. 2025 Jan 19:2025.01.18.25320774. Preprint. [Version 1] doi: 10.1101/2025.01.18.25320774 (PMC11759617; doi:10.1101/2025.01.18.25320774)
Supplement: Supplementary file 2 [file media-2.pdf]

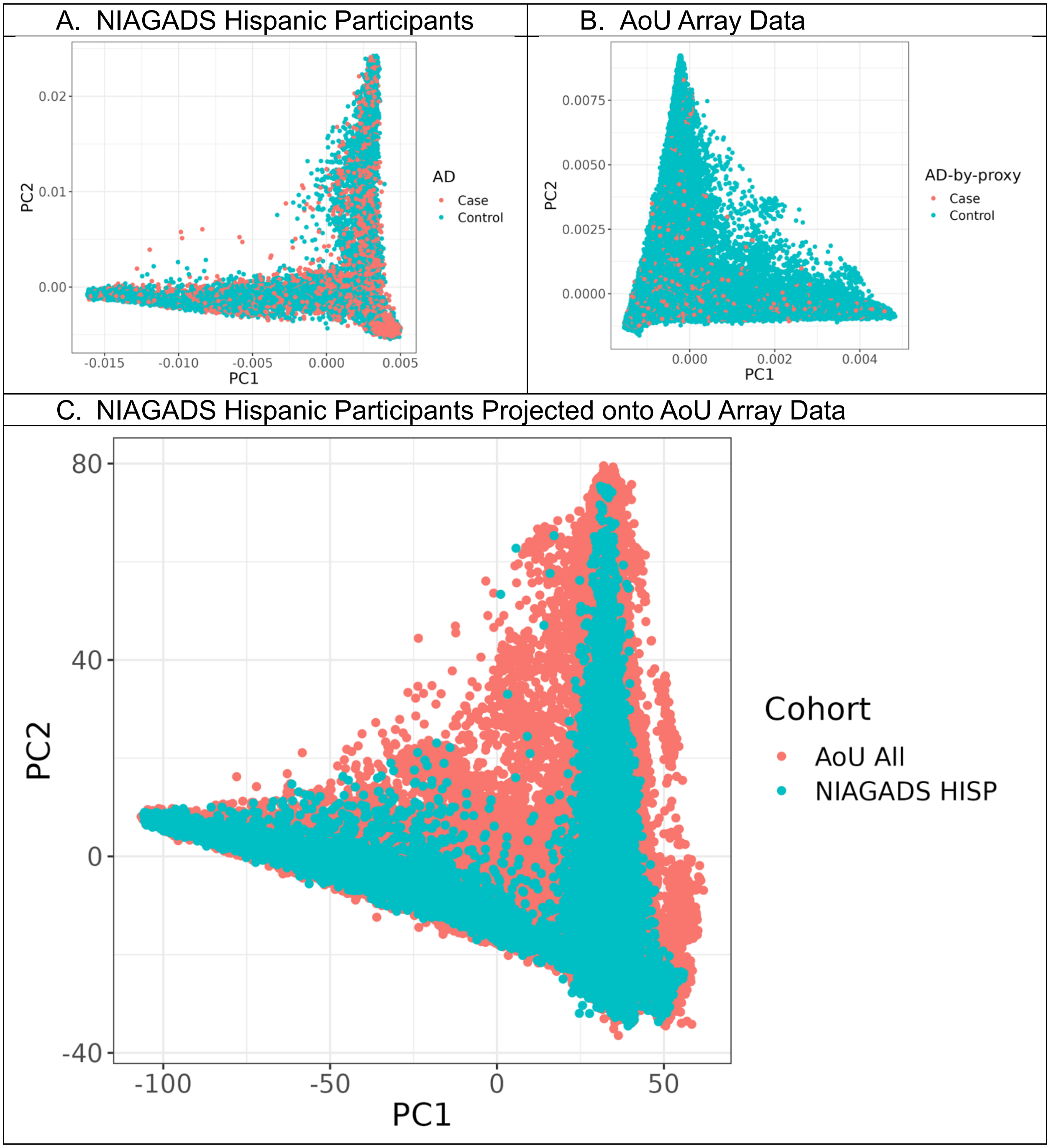

Supplemental Figure 2. Principal component plots for each dataset
