# Supplementary material for: Matching Heterogeneous Cohorts by Projected Principal Components Reveals Two Novel Alzheimer’s Disease-Associated Genes in the Hispanic Population
Source: medRxiv. 2025 Jan 19:2025.01.18.25320774. Preprint. [Version 1] doi: 10.1101/2025.01.18.25320774 (PMC11759617; doi:10.1101/2025.01.18.25320774)
Supplement: Supplementary file 3 [file media-3.pdf]

Supplemental Table 1. Demographic information for each cohort in our study. Single ancestral population percentages represent the proportion of the total sample size composed of the indicated ancestral population.

Cohort	**NIAGADS Hispanic**	**AoU Matched by Gen PCs**	**AoU Matched by Gen PCs, Age, and Sex**	**AoU Hispanic**
Outcome	Clinical AD	AD-by-proxy	AD-by-proxy	AD-by-proxy
Cases	2,559	9,479	4,633	1,695
Controls	5,908	35,314	20,869	54,868
Total Sample Size	8,467	44,793	25,502	56,563
Case Percentage	30.2%	21.2%	18.2%	3.0%
African Ancestry	1,528 (18.0%), 628 Cases	2,130 (4.8%), 996 Cases	3,175 (12.5%), 615 Cases	5,650 (10.0%), 85 Cases
Admixed-American Ancestry	1,356 (16.0%), 264 Cases	32,855 (73.3%), 1,100 Cases	15,674 (61.5%), 767 Cases	43,328 (76.6%), 1,056 Cases
European Ancestry	3,721 (43.9%), 1,402 Cases	7,142 (15.9%), 7,077 Cases	5,174 (20.3%), 3,097 Cases	7,042 (12.4%), 538 Cases
Asian (East or South) Ancestry	2,065 (24.4%), 329 Cases	2,551 (5.7%), 285 Cases	1,431 (5.6%), 146 Cases	485 (0.9%), 14 Cases
Middle-Eastern Ancestry	NA	115 (0.3%), 21 Cases	48 (0.2%), 8 Cases	58 (0.1%), 2 Cases
Age: Mean (SD)	71.7 (9.6)	50.2 (16.4)	67.9 (13.4)	50.2 (16.3)
Female Sex Percentage	67.2%	66.5%	66.0%	62.1%
rs429358 MAF (ApoE4)	14.5%	12.8%	12.8%	12.8%
rs7412 MAF (ApoE2)	4.2%	4.9%	5.6%	5.3%
